# The Use of the Clinical Ethnographic Narrative Interview to Understand and Support Help Seeking After Gender-Based Violence

**DOI:** 10.4473/TPM24.3.8

**Published:** 2017-09

**Authors:** Denise M. Saint Arnault

**Affiliations:** University of Michigan School of Nursing

**Keywords:** Narrative interviewing, Women's mental health, Trauma recovery, Meaning after trauma, Self-discovery

## Abstract

Gender-based violence (GBV), characterized by the abduction or rape of women and girls to humiliate, intimidate, and traumatize them and their communities, is a profoundly disturbing tactic in international conflict. Long after armed conflict has ended, survivors continue to experience physical injuries, psychological trauma, and social and cultural stigma. Guilt, shame, and continued interpersonal violence can become a normalized part of daily life, significantly challenging the road to healing and recovery. Research about self-disclosure and narrative after GBV has shown that help seeking rates are shockingly low, with estimates ranging from 4-27%. From a feminist and a humanistic perspective, studying trauma history and related help seeking is delicate work that must use interview processes that ensure the survivor can tell her story without revictimization, while also aiming to restore personal mastery, empowerment, and self-understanding. Based on theories about benefits and challenges of the narrative after GBV and trauma, we propose that the Clinical Ethnographic Narrative Interview (CENI) allows researchers and practitioners a safe container to examine the complex interplay between suffering, culture, and help seeking. Using this interview, the interviewer and the participant work as partners to define, compare, and contrast the socio-cultural barriers and facilitators of help seeking. This paper explains the narrative theory and the challenges and benefits of the narrative approach after trauma. Then we provide support for the use of the CENI for an understanding of the help seeking process and facilitating a health-promoting narrative interview for survivors. We then address implications for research, practice, and policy.

The experience of pain is a matrix of meanings, identities and relationships situated in a particular culture (Bakan, 1968, p.57)

Gender-based violence (GBV) can be difficult for survivors long after the conflict has ended, and the healing journey may include persistent physical complaints, psychological trauma, as well as social and cultural stigma. Guilt, shame, and continued interpersonal violence can become a normalized part of daily life, significantly challenging the road to healing and recovery (World Health Organization, 2016).

Healing and recovery for these women require social engagement, yet surprisingly little scholarly attention has been paid to this kind of help seeking. Research has indicated that narrative self-disclosure can be therapeutic, and self-disclosure is the gateway to help seeking for trauma recovery (Androff, 2012; Herman, 1997; Stover, 2004; Wood & Roche, 2001). Research has suggested that general practitioners, family, and friends are the preferred choices for help seeking (Ansara & Hindin, 2010). However, women are reluctant to disclose a trauma history even from these sources. Only one-third of Canadian women sampled from general practice office who had experienced violence within the last year reported their stories to their practitioner (Barrett & St. Pierre, 2011). Cultural and social stigma combined with shame prevents women from disclosing their trauma. In a study aiming to understand factors that prevent help seeking, Fugate, Landis, Riordan, Naureckas, and Engel (2005) interviewed 491 women with a history of violence from primary care services, finding that only between 20-35% contacted any formal source, and 29% did not talk to anyone about the violence. One New Zealand study showed that of the 700 women who reported disclosing the violence, 40% said that no one had helped them (Fanslow & Robinson, 2010). Finally, in a multi-national survey of 42,000 women across 28 European Union Member States, the Fundamental Rights Agency found that help seeking rates ranged widely, depending on the types of services and the country. For example, rates for contacting police were lowest in Finland at 7%, and the highest in Cyprus at 27%. However, rates for contacting victim support services such as rape crisis centers or domestic violence agencies ranged from 0 to 13%. The most widely used source was medical services, at 2% in Greece and 33% in Belgium (European Union Agency for Fundamental Rights, 2014).

From a feminist and a humanistic perspective, scholars and practitioners want to help women reveal trauma as the first step toward healing, however, we can see that women are extremely reluctant to do so. Research and intervention must to ensure that the survivor can tell her story without revictimization, while also aiming to restore personal mastery, empowerment, and self-understanding. This article examines theoretical and empirical research on the complexities of narrative intervention for trauma recovery, finding that the investigation about the benefits and potential harm in the process is mixed. We theorize about what a narrative is, how the process is interrupted by trauma, and explore the factors within a narrative that can be healing. Next, we review the development of the Clinical Ethnographic Narrative Interview (CENI) as a format that facilitates sense-making, personal control, and empowerment by providing the healing components of narrative, which include articulating and organizing experience, as well as finding meaning in life events for women who are in the process of healing for psychological and trauma related distress (Saint Arnault & Shimabukuro, 2012). These keys to recovery inform real healing in the future, promoting self-understanding, help seeking, and healing.

## The Nature of the Narrative

The narrative is the mental activity that consciously organizes the unifying links between the self, relationships, time, and morality (Crossley, 2000). The narrative is an essential part of the fabric of social exchange. Narrative constructions are human acts of sharing stories, providing ways to relate together and validate each other (Wigren, 1994). Several psychological elements combine to become a narrative. First, stories attend to experienced sensations. Next, narrative involves a cognitive-perceptual interpretation process of the relevance of internal and external elements of the environment to the felt sensation. Then, the story develops causal chains that locate events as causes or consequences of other incidents. Narrative connects events and characters in ways that both evoke and account for sensation and feelings. Narrative organizes events episodically, dividing the stream of consciousness, linking individual experiences together, while separating them from others. The connections between the sensations, the actors involved, and the causes and consequences of the events create meaning, understanding, and insight. The conclusions drawn from these definitions guide future behavior and contribute to the ongoing formation of a worldview and a personal identity. Therefore, narratives provide and contain the understandings that permit the use of experience in understanding, predicting, and responding to future experience (Schank, 1990).

## Trauma, Shame, and Narrative

The word “trauma” has its origin in the Greek word *trauma*, which refers to a wound. Trauma disrupts sequential processing by interfering with psychophysiological coordination, cognition, memory and emotional processing and storage, and social connections after trauma. The stories formed during trauma are frequently incomplete, especially regarding higher order elements, including environmental recall, temporality, and causal linkages (Wigren, 1994). At the experiential level, this incomplete recollection introduces self-doubt and shame within the victim, inhibiting self-disclosure and the social exchange necessary for the construction of the personal narrative. Because narrative involves connections between thoughts, feelings, context, relation- ships, and meanings, the narrative can be said to be the *construction of the self through language*. However, because trauma narratives require the person to “recall” the traumas they experienced, this construction involves the re-experiencing of distressing emotional and sensory states, which are often void of time, sequence, and context. This psychophysiological condition can make the trauma experience feel chaotic, overwhelming, dangerous, unpredictable, and unexplainable. The nature of this disruption and fragmentation creates a kind of memory incoherence, which contributes to the challenge of rebuilding the individual's shattered sense of identity and meaning that can be achieved through narrative (Crossley, 2000). From a social-selfhood point of view, this makes the recollection and narrative of trauma an unclaimed and unarticulated set of experiences which remain silenced and unheard, disabling help seeking and recovery (Bakan, 1968).

As we have seen, impairment of memory and recall during and after traumatic events can affect the survivors' ability to tell their story and make meaning of their lives. This notion of memory encoding requires a brief look at neurobiological processes during and after traumatic events. First, there are two kinds of memory. Explicit memory or factual memory is a consciously accessible repository of experience, containing one's perception or sense of self during that time in one's life (Minshew & D'Andrea, 2015; Siegel, 2008). Narrative production relies on this kind of memory because this is where details, time, and chains of cause and effect are understood. Some research refers to explicit memory as working memory, as well as executive functioning, and it is said that memories that are encoded here are accessible because they are in the area of the brain that also contains language, cause and effect processing, and decision-making (Siegel, 2008).

However, some research has suggested that when someone experiences a terrifying event or is overwhelmed by emotion, the memories that are stored are primarily implicit memory. Implicit memories are sometimes referred to as “body memories, or “non-declarative memory,” emphasizing that associated language is often absent, and that these memories have not yet been connected to the higher order executive functions of language and logic (Minshew & D'Andrea, 2015; Van der Kolk, 2015). Implicit trauma memories come unbidden to the survivor, and are experienced in the form of bodily sensations, visual fragments, dreams, and “unexplainable” feelings of anxiety or terror (Siegel, 2008). Some research has suggested that one of the values of developing narrative after trauma is the “lifting” of memories out of the implicit realm of the mind, and into the explicit or executive functions of the brain for storage and conscious processing (Koch, Fuchs, Summa, & Müller, 2012).

Shame often accompanies trauma. Shame is an intensely negative emotion, fueled by the experience of being a “broken self” (Herman, 2011). Shame has to do with personal standards, responsibility, and failure, especially when one perceives the failure as a personal responsibility. Shame is the self-in-the-eyes-of-the-other (Bockers, Roepke, Michael, Renneberg, & Knaevelsrud, 2016). Shame is especially present in relationships of subordination and control, generating a family of emotions such as humiliation, disgrace, dishonor, and self-loathing. Shame, like trauma, includes physiologic responses that overwhelm higher cortical functions, such as language, temporalizing, and decision making (Herman, 2011). In interpersonal and sexual trauma, these psychophysiological responses of shame compound the difficulty to create a narrative. Moreover, shame is an element of a narrative in which the victims blame themselves, therefore seeing themselves as a cause of the trauma. This feeling of shame is the belief about the self in the story; becoming a meaning construction that prompts actions that avoid being “found out,” or exposed, or intimate with others.

## Healing or Re-Traumatization from Narrative Disclosure

Narrative is a psychosocial vehicle that essentially involves one “telling their story.” However, there is a wide variety of applications of using narrative as a technique with survivors of violence. One form is testimony narratives, which are used in reconciliation courts to provide victims with the valued opportunity to tell their story to the wider world (Stover, 2004). Another form is the psychotherapeutic use of narrative as an intervention to promote healing and recovery (Wood & Roche, 2001). In general, we might refer to these diverse applications as a narrative technique that provides a structure or container through which the victim will disclose sensitive information. From a psychotherapeutic perspective, narrative techniques, when provided by a validating third party (whether it be within a hearing, provided by a therapist, or carried out in a social support group setting), can bring relief from emotional and psychological pain, anguish and suffering, and promote coping with violent crime and other abuses (Mendeloff, 2009).

Because narrative has been employed in a range of violence types (e.g., genocide, torture, political repression) and in a variety of international settings (Androff, 2012), it is important to understand efforts to evaluate the therapeutic value of these techniques. The use of narrative techniques as a psychotherapeutic intervention to promote recovery (Herman, 1997) has generally been referred to as “narrative therapy,” and has been described as therapeutic when victims' previously silenced voices are received with deep listening that authenticates and validates abuse by bearing witness (Wood & Roche, 2001). In one empirical study of the use of narrative therapy for Chilean victims of imprisonment and torture, individuals experienced cathartic benefits as well as relief of symptoms of anxiety and depression (Cienfuegos & Monelli, 1983). Neuner, Schauer, Klaschik, Karunakara, and Elbert (2004) examined Narrative Exposure Therapy (NET) which they described as a short-term approach based on cognitive-behavioral therapy and testimony therapy. In a randomized controlled trial with Sudanese refugees living in Ugandan refugee settlement (*N* = 43), they compared the NET group with supportive counseling group (who received psychotherapeutic support for coping and catharsis, but did not receive narrative techniques) and a psychoeducational group (who received and discussed information about trauma). They found that significantly fewer participants in the NET group had post intervention post-traumatic stress disorder (PTSD) compared with either the supportive counseling group (*p* = .01) or the psychoeducational group (*p* = .02). Unfortunately, these trials do not explain or measure the mechanisms that can account for the variation in responses to the therapy, making an evaluation of these findings difficult. However, Neimeyer and Stewart (1996) suggested that when narrative emphasizes accountability, it is more likely to be healing.

Another aspect of narrative techniques that has received some evaluation is the impact of truth-telling. Observers have warned that an emphasis on truth-telling, without the therapeutic framework, can be counterproductive or re-traumatizing for victims, especially when used for reconciliation and long-term peace in war-torn societies (Brounéus, 2008; Cerulli, Poleshuck, Raimondi, Veale, & Chin, 2012). For example, a disturbing analysis of in-depth interviews with 16 Rwandan women in village tribunals found that they experienced re-traumatization, ill-health, isolation, and insecurity before, during, and after their testimony (Brounéus, 2008). Cerulli et al. used focus groups with 31 women in domestic violence courts. They found that daily experiences of body pain from abuse triggered emotional and psychological pain, and that constant court battles related to parenting and prosecution efforts increased participants' exhaustion, frustration, and re-traumatization. This literature does not measure the variables that are necessary to protect victims, nor what factors are necessary to promote health.

Taken together, these findings reveal that narrative is a critical psychosocial act that is complicated, or perhaps disabled by the interacting dynamics of the trauma experience and the belief in personal responsibility or shame. Available literature does not provide standards regarding about how best to facilitate narrative techniques in such a way that it is healing, and there is concerning research suggesting that the act of “telling the story” may not, in itself, be enough to promote healing. There is a suggestion that narrative needs to be facilitated by the presence of a sensitive witness, especially when this witness provides validating responses. It is possible that helping the development of a narrative that assigns appropriate accountability for the events, can help healing. Finally, since narratives are about meaning making, it is possible that helping women craft an empowerment narrative will be helpful. However, psychophysiological responses to trauma and shame complicate narrative construction. Here, we propose that interventions that can facilitate women's ability to “fill in the gaps” of their implicit memory, examine the meaning of events in their lives, and that use this self-understanding to move into the future, may be necessary components of narrative healing. The aim of the Clinical Ethnographic Narrative Interview is to help the trauma survivors organize their experiences and the trauma contexts, and make sense of experience through examination of causes and meanings. Below, we explain the development of this narrative facilitation method and the theory that was used to develop each component.

## Method

### The Clinical Ethnographic Narrative Interview (CENI)

In early work with distressed Japanese women, the author used anthropological and narrative techniques to create a structured container that helped women explore social and cultural aspects of suffering, meaning, healing, and help seeking. Because the interview focused specifically on distress and suffering, we referred to it as a “clinical” interview. In addition, because we emphasized the social and cultural meaning-making of the suffering, we referred to it as an ethnographic interview. Therefore, we originally called our interview the Clinical Ethnographic Interview (CEI; Saint Arnault & Shimabukuro, 2012). In later thinking, we recognized that the central feature of the interview was the narrative techniques that facilitated telling one's personal story, and we have renamed the interview the Clinical Ethnographic Narrative Interview (CENI). This paper is informed from research using the CENI with distressed immigrant women (Saint Arnault & Fetters, 2011; Saint Arnault & Shimabukuro, 2012), but also from work carried out by an international consortium of researchers who have used the CENI with convenience samples of women who identified as having experienced GBV (USA, *N* = 22; Ireland, *N* = 12; Romania, *N* = 5; Italy, *N* = 5) (Saint Arnault et al., 2017). Women for this international study were recruited from domestic violence service centers, health services, and from social services agencies. All research materials were translated into the languages of the research site. All women received the CENI at a location of their choosing in their native language. All interviewers were trained by the author. The research was approved by University of Michigan Institutional Review Board (IRB). Most interviews lasted about 1.5 hour and were audiotaped.

The purpose of the CENI narrative format is to facilitate a participant to connect aspects of life together and to examine these connections from a variety of angles. The CENI was developed to help participants articulate the meaning in their lives and to relate that meaning to healing and recovery from a new, more aware vantage point. The CENI's narrative approach is an engaged process through which unconscious patterns of one's life are brought into consciousness, and can, therefore, become reorganized and integrated into consciousness (Gergen & Gergen, 1986). Because memory about traumatic experiences can be incomplete or unconscious, the CENI uses activities that begin as non-verbal drawings. Art renderings have been used for trauma therapies for children and adults for decades (Lu & Yuen, 2012; Meiring & Müller, 2010; Meyburgh, 2007), and some research is looking at the mechanisms for these methods for trauma recovery (Spiegel, Malchiodi, Backos, & Collie, 2006). The CENI process allows the participant to access non-verbal (implicit) memory first, then work in the interview to “put words to,” and organize experiences. The CENI narrative method uses holistic data that illuminates interactions among gender, culture, beliefs, social networks, resources, competencies and strengths, as well as dilemmas imposed by sociocultural constraints and traumatic experiences.

The CENI was constructed to illuminate patterns by facilitating an unfolding journey through one's experiences. The psychological journey of unfolding in the CENI is a movement from external to internal, then to chronological, and into experiential focus, integration, and evaluation, and then back out to one's life, and into the future. We begin with a *social network map* to explore the participant's current situation within her social context (external). Next, we invite the participant to use *body mapping* to place her distress onto her body (internal). Then, the participant completes a historical overview of her distress in a *lifeline*, linking situations, stressors, responses, and actions (chronological). Next, the *card sort* helps the participant describe and put words to current distress by creating an experiential map of feelings (focus). Finally, these four products are the basis for questions about cause, consequence, meaning, the self, and the future (integration, evaluation, and the future).

#### Social Network Map

The social network map is an introduction to the self-in-contact. The participant, while drawing her network map, considers her relationships, level of social engagement, her perception and use of social resources, as well as the perception of negativity and stigma. The participant then describes her understanding of who is a resource, what relationships feel conflictual and considers sources of social stress or support. This self-in-contact sets the stage for the rest of the interview, situating women within their personal, family, and community's expectations. Participants return to this picture throughout the interview, adding detail, and explaining their feelings about this social context.

#### Body Map

**Figure 1:**
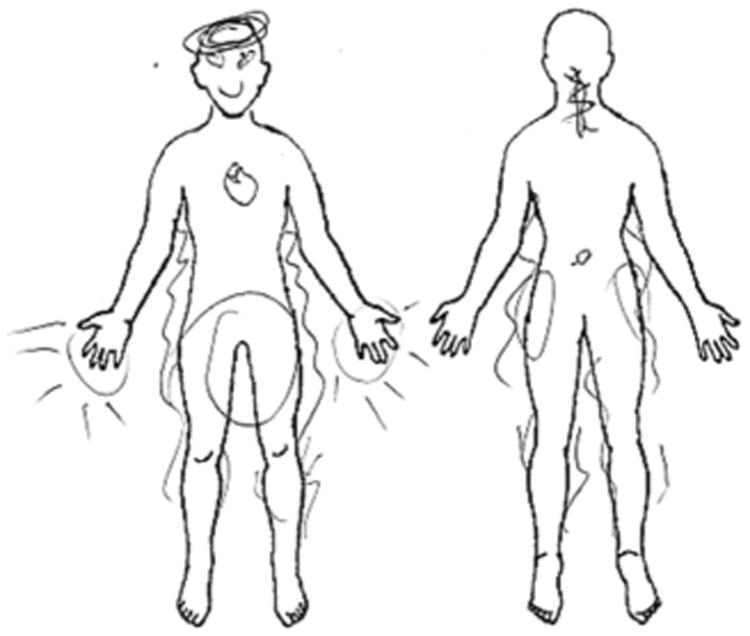
Body map.

The body map represents a movement from the externally oriented self to the internal self. The body map supports a process of personal reflection and an inwardly oriented awareness of the self (Brett-MacLean, 2009; Cregan, 2006; Evans, 2010; MacGregor, 2009). Body mapping involves the participant placing significant events, illnesses, distresses, and joys upon the body as a way of locating their impact in the physical, material, and experiential world. The body map not only helps the participant shift her focus toward the internal, but also reminds her to take the body seriously. The body map reveals current feelings (both positive and negative), as well as general daily life experiences, such as sleeping, eating, weakness, fatigue, and other symptoms that affect functioning (Figure 1, Participant A012; Saint Arnault, et al., 2017).

#### Lifeline

The lifeline facilitates the “creating order” function of the narrative, prompting the participant to gather themselves across time (Frank, 1984; Gramling & Carr, 2004; Shimomura, 2011; Taguchi, Yamazaki, Takayama, & Saito, 2008). The lifeline is not intended to be an “accurate” or “complete” representation of life events. It is what events stand out for the participant at the time of the drawing. In that way, the lifeline is a “compare and contrast” activity, in which the participant references events against what is “now,” as well as referencing events against each other.

**Figure 2:**
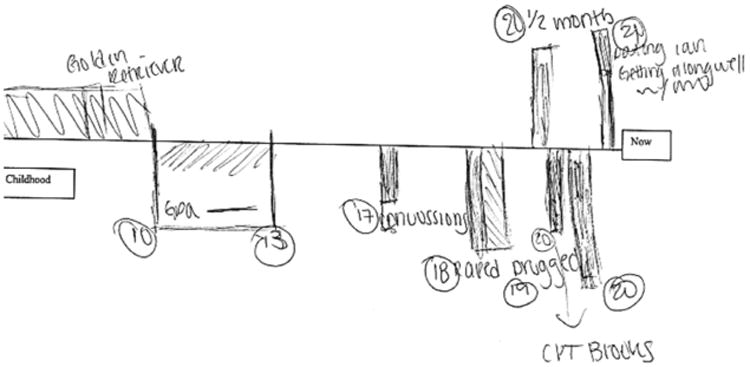
Lifeline.

The lifeline depicts highs and lows within the life of the women, revealing patterns of distress symptoms and efforts to seek help across time. Discussion of the lifeline usually develops the line with participants adding events, linking or contrasting events and feelings, and exploring similarities and differences of life events regarding meaning and context. Women examine how they dealt with stress or distress, and what worked and what did not. They begin to recognize how they moved in and out of low times, risks and stressors, or better times where they effectively used resources. During the lifeline, participants describe how events were perceived, and how these perceptions contributed to help seeking decisions (Figure 2, Participant A042; Saint Arnault et al., 2017).

#### Card Sort

**Figure 3:**
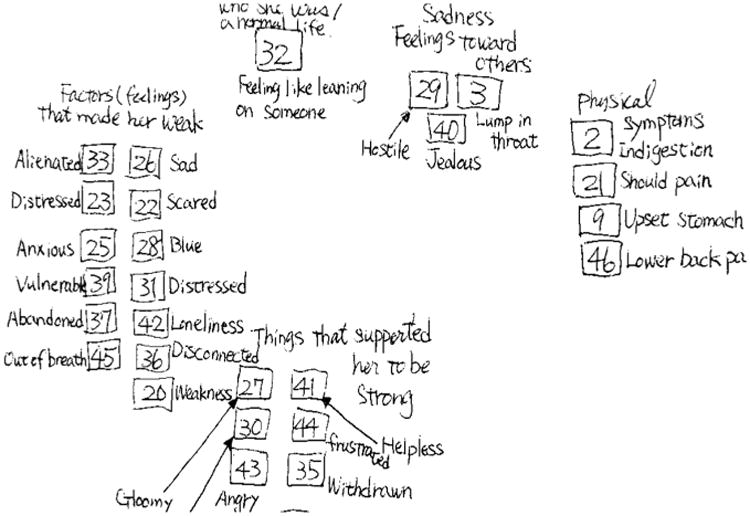
Card sort.

While the lifeline helps with organization of events and feelings, because it covers a vast array of experience, it is necessarily general and global. The lifeline gives the participant clues to how they felt at any given time and patterns of events, and during the process, participants will begin wondering about the details of specific incidents. In our opinion, however, while the process of self-awareness begins with the general, it is within the particular that people derive meaning. To finish the narrative, the process of the card sort is used to aid in focusing (Borgatti, 1999; Canter, Brown, & Groat, 1985; Fraser, Estabrooks, Allen, & Strang, 2009; Gordon, 2001; Neufeld et al., 2004). In the CENI, the participant is directed to focus on a recent point in life. We select low points because seeking help is about mitigation of suffering (Figure 3, Participant R481; Saint Arnault & Fetters, 2011).

The participants receive a stack of cards that include emotional and physical feelings that have been gathered from previous studies (Saint Arnault, 2008; Saint Arnault & Fetters, 2011; Saint Arnault & Kim, 2008; Saint Arnault & O'Halloran, 2014, 2016; Saint Arnault & Shimabukuro, 2012), and choose cards that describe or represent their low point. They cluster these feelings into whatever groupings are relevant and explore these groupings. Most often, women appear to “climb into” or experience these feelings while doing the card sort. The discussion of these feelings is highly sensitive because they are close to the surface. During this task, the interviewer does not ask about the circumstances that created the distress. Importantly, the interviewers aim here is to help the participant “make meaning” about these experiences by examining her beliefs during them, the meanings they held at the time, how that meaning affected her actions at that point.

#### Integration: Causes, Consequences, Meaning, and the Future

The final step in the CENI process is the integration of all the parts of the story together. While analyzing a focused event, the participant is engaged in “putting all of the pieces together” to make sense of the gestalt of the narrative, and to interpret causes, meanings, consequences, and the future. While the participant is “feeling into” the chosen experience, the interviewer facilitates interpretation of the feeling by helping the participant think about what caused them, what they meant at the time, what they mean now, and how this meaning has shaped themselves and their lives.

Finally, the interview concludes by helping the participant bring these meanings “back out,” connecting them onto the lifeline, the body map, and the social network. It is here that the comparison and integration process begins anew, because the participant brings forward deeper meanings and self-knowledge, mapping them onto aspects of their lives. During this integration, the participants are asked to describe a summation of who she is, what she knows about life, and how she plans to go forward.

## Discussion

The lifeline is an interesting experience that helps a person sequence events. We believe that it is the process of *constructing* the lifeline that is the therapeutic agent. The lifeline is not intended to be a historically accurate rendering of life events. It is likely that a lifeline drawing might feature different aspects of one's life at each rendering. Indeed, if memory fragmentation is a consequence of trauma, then we can expect that trauma survivors will have some memories characterized by implicit, non-declarative, and emotionally charged remembrances, experienced by the participants as missing pieces or gaps. Despite the challenge for the trauma survivor, the act of sequencing and organizing one's life is validating because one puts their “life” down on paper, giving it credibility and stability. Going through the lifeline process reveals both details and gaps, making it necessary psychological work for the survivor. After doing the interview, participants report that they want to think more about it. For example, one woman said as she completed her interview “I guess I do not know what I was doing during that time. I am going to have to give this some more thought.” We believe, like other memory researchers, that a beginning step toward self-knowledge is the act of combating fragmentation and memory gaps (Herman, 1997; Schore, 2009; Siegel, 2008; Van der Kolk & Fisler, 1995).

In the development of the CENI, the author relied heavily on clinical experience as a feminist, a psychotherapist, and an anthropologist. Feminists hold the ethical mandate to let a woman tell her story while promoting empowerment and self-mastery. Training as a psychotherapist helped with understanding about how to facilitate and trace the psychological unfolding of painful memory, as well as how to help people come to closure in a single session. Finally, anthropological training suggested ways to help uncover implicit memories, which are the source of beliefs, motivations, and meaning. The purpose of the interview was to provide a safe container within which a participant could move through a predictable process that ended with an enhanced sense of self-understanding, as well as a plan to go forward. This interview was designed to be self-contained, but also to support the participant's access to their emotions and their interpretations about their painful history. Therefore, it is critical that the psychological movement of the participant's experience be carefully facilitated to help them bring forward any new awareness they gain during the lifeline and card sort. Understanding this process can be facilitated with an allegory: the participant and the interviewer-companion go underground together, find an important treasure, and come back out together to decide where she should put it in her treasury. The participants need time and support to articulate what they have learned, what that means to them and for their lives in order to grow in health and integrity. This act of making what is tacit conscious, and integrating that into one's whole being is, in essence, the psychological achievement of the narrative and the goal of narrative therapy (Androff, 2012; Bakan, 1968; Herman, 1997). This process is also one's search for meaning (Frankl, 1985), which is the goal of meaning-centered therapies such as logotherapy (Southwick, Lowthert, & Graber, 2016).

We do not yet fully understand the therapeutic mechanisms through which the artistic renderings produced in our interview may facilitate trauma recovery. Some research suggests that the act of drawing may stimulate alpha rhythm brain waves, which may be associated with implicit memory retrieval, visual processing, and emotional self-regulation (Belkofer, Van Hecke, & Konopka, 2014). In the Belkofer et al.'s study, EEG recordings showed differences the alpha frequencies for the pre- and post-drawing samples. The drawings used in our study (social networks, body maps, lifelines, and card sorts) may not be the same as the products developed in other kinds of artistic production, such as painting or sculpture. Nonetheless, women in our studies have remarked that they recognize new aspects of their lives and understand more about themselves and their lives through these various processes. Certainly, if trauma memories are encoded as implicit or non-declarative (i.e., non-verbal), then methods that help with memory retrieval and integration may be beneficial. Since a narrative relies on explicit memory, any means that enhances access to non-declarative memory and the articulation of it in a safe space seems useful. However, additional research is necessary to understand the full scope of these methods for recovery.

The role of the interviewer is vital in narrative therapy in general, and the CENI is no exception. In preparation for carrying out the CENI, we train interviewers to take the stance of a “curious witness.” Their role is to support the flow of the interview so that it is a safe container for the participant to process their experience. In this way, it is critical that they understand that flow and the psychological underpinnings of it. It is also essential that *the participant* creates the interpretations, associations, and connections between the products. This caveat is central to the psychological safety of the encounter. The survivor will connect and interpret whatever she is psychologically ready to process and, therefore, has complete mastery over the depth of the process. This mastery is an empowering position for the survivor and may be one of the psychological benefits of the process. Also, like in other narrative approaches, deep listening can be, in it-self, healing, because it is validating (Wood & Roche, 2001). While the interviewer does not interpret material, he/she is, even so, in the critical position to track material across the different domains of the interview. For example, sometimes, in the interpretation of meaning in the interview, the participant might bring up the name of a person that they mentioned in their social network. We recommend in training that the interviewer simply indicates that social network person. Alternatively, a participant may mention a connection to the focusing event (card sort) and a different event on their lifeline. Again, the interviewer is trained to simply point to that event on the line. This subtle cueing enables connections that *the participant makes* with their drawings. It concretizes the connections and demonstrates coherence and meaning between the products. This meaning and its integration is the aim of the interview.

We have seen evidence that the CENI can assist women in organizing and articulating meaning about their low points in several studies (Hatashita et al., 2015; Saint Arnault & Fetters, 2011; Saint Arnault & Roels, 2012; Saint Arnault & Shimabukuro, 2012, 2016). However, additional research is necessary to determine whether the CENI provides long-term benefits. The efficacy of the CENI to promote health and healing is currently being researched. For the women in the GBV study, the women in the American sample evaluated the value of the CENI to help understand meaning, look at things in a new way, and to provide self-awareness. Items were scored 0 for *not at all*, 1 for *somewhat*, and 2 for *fully*. The average rating was 9.3 out of a possible maximum score of 14 (Saint Arnault et al., 2017). Also, substantial work remains to isolate the mechanisms through which these approaches help recovery. The CENI was created to facilitate help seeking on the theoretical assumption that seeking help occurs within a social context, and that help seeking actions are based on the interpretation of sociocultural and intrapersonal meanings (Saint Arnault, 2009; Saint Arnault & O'Halloran, 2016). What we assert here is that the help seeking can be disabled in trauma recovery and that promoting self-awareness and meaning-making through facilitating narrative self-disclosure is empowering, enables self-mastery, and supports trauma recovery, decision making, and action.

The limitations of the CENI are that it requires careful and repeated training to help interviewers to understand the difficulties that survivors may have in constructing their narrative, and to help the participants explore the various aspects of the interview vis-à-vis each other. However, this kind of sensitivity and training is necessary in all research with violence survivors. Some participants are initially intimidated with producing drawings rather than simply explaining, however, they generally find that the open-ended nature of the activities helps them look at their lives in new ways. Because of the impact of trauma on conscious recall, women may struggle to organize life events on the lifeline. However, healing, from the narrative perspective, rests on their ability to make the incoherent coherent, so the efforts to work on the lifeline help women to begin that process. In general, interviewers find that the interview is a powerful tool and our aim is to expand the use of the activities into more than one session, and perhaps working in groups. Additional research is needed to evaluate the impact of this interview compared with other formats, and to evaluate the impact of the interview on health outcomes important for survivors of trauma.
